# Alginate-Based Hydrogel Bead Reinforced with Montmorillonite Clay and Bacterial Cellulose-Activated Carbon as an Effective Adsorbent for Removing Dye from Aqueous Solution

**DOI:** 10.3390/gels10090597

**Published:** 2024-09-16

**Authors:** Muhammad Dody Isnaini, Bhawaranchat Vanichsetakul, Muenduen Phisalaphong

**Affiliations:** Bio-Circular-Green-economy Technology & Engineering Center, BCGeTEC, Department of Chemical Engineering, Faculty of Engineering, Chulalongkorn University, Bangkok 10330, Thailand; muhammad.dody06@gmail.com (M.D.I.); 6671009121@student.chula.ac.th (B.V.)

**Keywords:** bacterial cellulose, activated carbon, hydrogel beads, methylene blue, adsorption isotherm, reusability

## Abstract

According to environmental concerns related to water pollution, this study aims to develop a novel hydrogel bead as a biocompatible and efficient adsorbent by integrating bacterial cellulose-activated carbon (BCAC) and montmorillonite (MT) in alginate hydrogel (ALG). The ionotropic gelation method was applied to the fabrication of BCAC/MT/ALG hydrogel beads. The BCAC/MT/ALG hydrogel bead exhibited significantly higher tensile strength, Young’s modulus, and thermal stability, with ~1.4 times higher adsorption uptake of methylene blue (MB) from aqueous solution as compared to the pristine ALG bead. The textural properties, including specific surface area and porosity, were beneficial to accommodate the size of cationic MB as the target molecule. This resulted in a remarkable MB adsorption uptake of 678.2 mg/g at pH 7 and 30 °C. The adsorption isotherm showed the best fit for the nonlinear Redlich-Peterson isotherm model. Experimental adsorption data were well-described by the pseudo-second order kinetic model, with *R*^2^ values reaching 0.997. In addition, the adsorbent bead demonstrated easy regeneration with high reusability with approximately 75% of MB removal after being used for six cycles. Therefore, BCAC/MT/ALG bead represents an eco-friendly, cost-effective, and highly efficient adsorbent for MB removal from water and could potentially be used for removal of a wide range of cationic dye pollutants from wastewater.

## 1. Introduction

Water pollution, exacerbated by global water resource scarcity, is a critical concern. Managing waterborne pollution, especially from organic dye discharge in sectors like textiles, pharmaceuticals and medical applications, paper, food, and polymers, has become increasingly vital [1,2,3]. Approximately 12–15% of dyes from the textile sector are released into the environment, leading to highly pigmented effluents [4,5]. This discharge poses an environmental concern due to its absorption and reflection of sunlight in water bodies, which reduces algae photosynthesis and negatively impacts aquatic ecosystems and biodiversity [5,6]. Among the various dyes employed, methylene blue (MB) has been widely applied in medical diagnostics and treatments, biological staining, chemical indicators, and as a synthetic dye for dyeing fabrics in the textile industry [7,8,9]. Inhaling this compound induces respiratory distress, and direct contact can cause permanent ocular damage, skin irritation, gastrointestinal discomfort, and mental health disorders [10,11]. Numerous technologies, including membrane separation, flocculation-coagulation, sedimentation, precipitation, and aerobic/anaerobic treatment, aim to reduce dye content in wastewater. However, most of these technologies might encounter feasibility limitations within industrial scales due to elevated operational costs, diminished efficiency, and environmental impacts [12,13]. Adsorption stands out among these approaches for its inherent benefits, including effective removal, simplicity in operation and handling, minimal sludge generation, ease of adsorbent regeneration, and cost-effectiveness [1,14].

Hydrogels with three-dimensional porous architectures have attracted significant interest as hydrogel-based sorbents due to their plentiful raw materials, cost-effectiveness, and high adsorption uptake. Using hydrogel beads as sorbents in wastewater treatment is advantageous due to their ease of separation, recycling, and being eco-friendly [15,16,17]. Sodium alginate, an anionic copolymer in a linear structure, represents a naturally occurring polysaccharide wherein β-D-mannuronic acid units are linked with α-L-guluronic acid units at the 1–4 position [18,19]. Sodium alginate, typically sourced from marine brown algae such as *Sargassum* spp., *Macrocystis pyrifera*, and *Ascophyllum nodosum* [18,20], is commonly used to increase the viscosity of aqueous solutions. Alginate or alginate composite beads can be crosslinked by exchanging sodium ions within guluronic acid residues with divalent cations such as Ca^2+^, Sr^2+^, and Ba^2+^, resulting in hydrogel formation through ionotropic gelation [12,21,22]. Sodium alginate is characterized by a range of functional groups, including hydroxyl and carboxyl groups [13,23]. It exhibits advantageous properties such as biodegradability, non-toxicity, and cost-effectiveness [24,25]. However, since sodium alginate beads demonstrate modest mechanical and low thermal resistance, the utility for large-scale applications is quite limited [20,26]. Integrating materials, including graphene, silica, biochar, and activated carbon, into alginate gel have been studied to increase the mechanical properties, thermal properties, and adsorption uptake of alginate-based composite beads [15,27].

Cellulose, a linear polysaccharide composed of D-glucose units linked in unbranched chains by β-1,4-glycosidic linkages, is the most plentiful biopolymer on earth [28,29]. Bacterial cellulose (BC), synthesized by certain species of bacteria (mainly produced by *Acetobacter xylinum*, reclassified as *Gluconacetobacter xylinum*) in various culture media, is a high-purity cellulose devoid of lignin and hemicellulose. BC exhibits superior properties such as tunable surface chemistry, high specific surface area, 3D-porous network structure, high modulus of elasticity and tensile strength, high degree of polymerization, and biodegradability [30,31]. Thus, the use of BC as activated carbon is intriguing to study.

Activated carbon (AC) holds a prominent place in the realm of wastewater treatment due to its extensive application in the removal of pollutants [32,33], owing to its substantial specific surface area, porous structure, and abundant surface functional moieties [2,33,34]. AC preparation involves two primary methods: physical and chemical activation. Chemical activation, preferred for its efficiency, operates at lower temperatures in a single step. Chemical activation agents such as H_3_PO_4_, H_2_SO_4_, HCl, and ZnCl_2_ are mainly employed [35,36], with H_3_PO_4_ being favored due to its environmental friendliness and effectiveness in developing porous characteristics [36]. However, AC powder in large-scale applications to the adsorption in wastewater treatment is challenging due to process complexities, high regeneration costs, and significant waste generation. Consequently, its utilization is partially limited and less favored [4,32].

Montmorillonite (MT), a member of the smectite group, is a dioctahedral aluminosilicate clay with a 2:1 clay mineral structure. It is characterized by the arrangement of two aluminosilicate sheets enclosing an octahedral alumina sheet [37,38]. This structural configuration leads to the development of a negatively charged interlayer space, primarily resulting from isomorphic substitutions, wherein ions such as Mg^2+^ or Fe^2+^ can substitute for Al^3+^. Within the interstitial layers of montmorillonite, a deliberate arrangement of hydrated cations such as Na^+^, Ca^2+^, K^+^, and Li^+^ serves the vital purpose of offsetting the prevalent negative charge [39,40]. Montmorillonite offers valuable properties for composite material development, including high specific surface area, mechanical durability, and eco-friendliness [14].

In this study, alginate-based hydrogel beads of BCAC/MT/ALG were fabricated by encapsulating bacterial cellulose-activated carbon (BCAC) and montmorillonite (MT) within an alginate (ALG) matrix. An efficient encapsulation was facilitated by cross-linking with calcium chloride. Using hydrogel beads as sorbents in water and wastewater treatment is beneficial due to their ease of separation, recycling, and low secondary pollution to address the limitation of powder adsorbent. In this study, a multi-component system based on alginate (ALG), montmorillonite clay (MT), and bacterial cellulose-activated carbon (BCAC) was applied for the preparation of the hydrogel bead to improve the capacity for removal of pollutants from water as well as to provide the possibility of regeneration of the system. The addition of fillers, BCAC and MT into the hydrogel of ALG offers important benefits for the improvement of surface area and strength of the materials. The characterization of the composite beads was conducted using Fourier-transform infrared spectroscopy (FTIR), nitrogen physisorption, thermogravimetric analysis (TGA), mechanical testing (UTM), and field-emission scanning electron microscopy (FE-SEM). The adsorption efficiency of BCAC/MT/ALG hydrogel beads was assessed using cationic methylene blue (MB) and compared with ALG and BCAC/ALG beads. A comprehensive analysis was conducted on adsorption isotherms, kinetics, and reusability studies.

## 2. Results and Discussion

### 2.1. Characterization of Adsorbents

#### 2.1.1. FTIR Analysis

**Figure 1:**
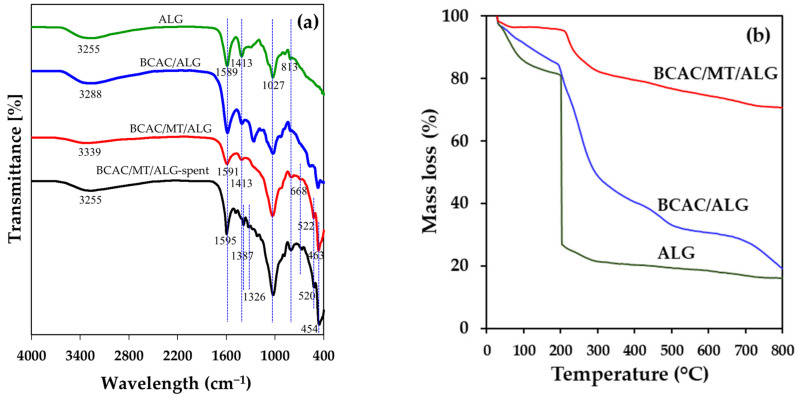
FTIR spectra (**a**) and TGA curve (**b**) of alginate and composite hydrogel beads.

The FTIR spectra presented in Figure 1a reveal distinctive absorbance bands spanning the range of 3500–3200 cm^−1^, which were attributed to the stretching vibrations of O−H bonds. Peaks observed at 1695–1586 cm^−1^ were indicated as a carboxylic group, signifying stretching vibrations of C=O bonds [33]. The bands at 1030–1025 cm^−1^ and 1408–1413 cm^−1^ were associated with the stretching vibrations of C−O−C/C−O bonds within the carboxyl groups of glycosidic linkages in alginate polysaccharides [32,41]. The peak at 1261 cm^−1^ indicated an asymmetric stretching vibration related to the C-O bond [42,43]. The characteristic peak of BCAC/MT/ALG at 799 cm^−1^ and the shifted peak after the adsorption process to 803 cm^−1^ were the Si-O-Si bending vibrations [44]. Distinctive bands at 522–520 cm^−1^ and 669 cm^−1^ corresponded to the bending vibrations of Si–O–Al bonds for octahedral Al. Bands at 463–454 cm^−1^ were indicative of the bending vibrations of Si–O–Si bonds, characteristic of montmorillonite [9]. Notably, new peaks emerged in the spectra at 1387 cm^−1^ and 1326 cm^−1^, attributed to the stretching vibrations of C=N bonds within the heterocyclic ring and the stretching vibration of N−CH_3_ bonds, both of which were characteristics of MB in hydrogel beads after the adsorption process [41]. The investigation displays slight shifts in wavelengths of fresh BCAC/MT/ALG as compared to the spent BCAC/MT/ALG, attributed to the interaction between the adsorbent’s surface functional groups and MB molecules due to electrostatic interactions and the formation of hydrogen bonds (H-bonds). The results from FTIR spectroscopy verified that the active sorption sites were associated with carboxyl and hydroxyl functional groups [4,23,33].

#### 2.1.2. Thermogravimetric and Mechanical Properties Analyses

Thermogravimetric analysis (TGA) was used to measure the mass changes of the samples over a temperature range from 30 to 800 °C, in order to observe the degradation behavior of the hydrogel beads for further applications as depicted in Figure 1b. The initial mass loss, occurring below 150 °C, was attributed to the evaporation of surface moisture and adsorbed water. ALG beads exhibited a sudden mass loss at around 200 °C, which was associated with the thermal degradation of alginate glycosidic linkages and the decarboxylation and decomposition of diverse organic compounds [9]. Whereas BCAC/ALG beads exhibited a slow mass loss between 200 and 800 °C, and slightly better thermal stability, for ALG and BCAC/ALG beads, the total mass losses at 800 °C were approximately 83.9% (residual mass of 16.1%) and 80.7% (residual mass of 19.3%), respectively. In comparison to ALG and BCAC/ALG beads, BCAC/MT/ALG beads exhibited gradual thermal degradation between 200 and 800 °C while maintaining a significantly higher residual mass of 70.9% (or 29.1% mass loss) at 800 °C, highlighting remarkable thermal stability improvements with MT incorporation.

Mechanical properties in terms of tensile strength, elongation at break, and Young’s modulus were determined as an average of five specimens, as shown in Table 1. In comparison to ALG beads, the incorporation of BCAC into ALG significantly enhanced tensile strength and Young’s modulus, achieving approximately 2.1- and 4.4-fold increases, respectively, whereas the percentage elongation at break considerably decreased to 22.0% of that of ALG. The result indicated an increase in rigidity as quantified by Young’s modulus, which measured a material’s stiffness and resistance to deformation under stress, and a loss of flexibility was observed. Integrating both BCAC and MT into ALG (BCAC/MT/ALG) resulted in a moderated increase in tensile strength and Young’s modulus (1.2- and 2.2-fold increases, respectively) compared to ALG. In addition, the elongation at break for BCAC/MT/ALG was significantly higher than BCAC/ALG, indicating that BCAC/MT/ALG was more flexible than BCAC/ALG. The decrease in elongation at break was attributed to the increased rigidity, which restricted the material’s capacity to stretch and deform before it broke [45]. Adding BCAC to ALG produced more rigid and stiff composite materials with greater tensile strength and Young’s modulus. However, incorporating MT into BCAC/ALG slightly reduced the rigidity and stiffness of the composite and resulted in a more flexible material.

In this study, the incorporation of activated carbon (BCAC) and montmorillonite clay (MT) improved the mechanical properties of hydrogel beads. Porous-activated carbons have been widely used as reinforcement in the polymer matrix to improve mechanical properties. It was explained that polymers could enter the pores of AC particles and form 3D networks through pore bonding, which improved the tensile properties of the composites [46]. MT is composed of units made up of two silica tetrahedral sheets with a central alumina octahedral sheet. The result from FTIR spectra of BCAC/MT/ALG showed additional bands corresponding to Si-O-Si and Si-O-Al bonds, which are characteristic of MT. Previously, improving the mechanical properties and thermal stability of sodium alginate composite films through the incorporation of SiO_2_ was reported [47]. It was suggested that the improvement in tensile strength (TS) might be related to the strong interactions formed by the hydroxyl groups of SiO_2_ with the carboxylic groups of the film polymeric matrix, reinforcing the material at high filler concentrations. However, there was no significant influence in the TS when the nano-SiO_2_ concentrations below 10% were applied, which corroborated the possible plasticizing effect of nano-SiO_2_, supporting the large increase in elongation at break. This study also found that the introduction of BCAC into ALG significantly enhanced tensile strength and Young’s modulus of the composite hydrogel bead, while the introduction of MT into BCAC/ALG significantly improved elongation at break of the BCAC/MT/ALG hydrogel beads.

#### 2.1.3. Surface Area and Pore Size Analyses

**Figure 2:**
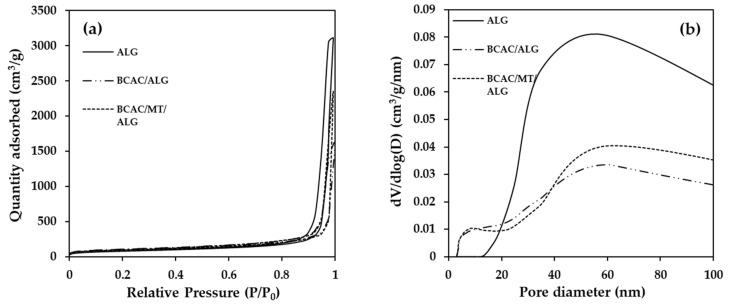
Nitrogen physisorption isotherms (**a**) and BJH pore size distributions (**b**) of ALG, BCAC/ALG, and BCAC/MT/ALG hydrogel beads measured at a temperature of −196 °C.

Nitrogen physisorption analysis was conducted to assess the BET surface area, pore volume, and pore size distribution of ALG, BCAC/ALG, and BCAC/MT/ALG beads, as detailed in Table 2. It shows that pure ALG beads exhibit a porous structure, featuring a total pore volume of 4.83 cm^3^/g, an average pore size diameter of 67.3 nm, and a specific surface area of 287.0 m^2^/g. The incorporation of BCAC and MT into ALG resulted in a reduction in the total pore volume of ALG beads. However, there was an apparent increase in a specific surface area of 35.2% and 16.9% higher for BCAC/ALG and BCAC/MT/ALG hydrogel beads, respectively. Additionally, Figure 2a presents the nitrogen physisorption isotherm profiles of ALG, BCAC/ALG, and BCAC/MT/ALG beads, displaying a type IV isotherm as the IUPAC classifies. This type demonstrates a significant and abrupt rise in N_2_ adsorption with a large hysteresis loop at high relative pressure (*P/P*_0_) ranging from 0.77 to 0.98, particularly for ALG beads. However, hysteresis loops were observed at lower *P/P*_0_, ranging from 0.52 to 0.99 for BCAC/ALG beads and from 0.47 to 0.98 for BCAC/MT/ALG beads. This indicates that ALG beads possess a greater quantity of mesoporous structure compared to BCAC/ALG and BCAC/MT/ALG beads. Furthermore, the H1 hysteresis loop identified in ALG beads indicates a confined spectrum of uniform mesopores, whereas BCAC/ALG and BCAC/MT/ALG hydrogel beads display both H1 and H3 hysteresis loops. These classifications are linked to non-rigid aggregates of plate-like particles of MT, contributing to a pore network comprising meso-macroporous structures incompletely saturated with pore condensate. The H3 hysteresis loop is characterized by a steep fall in the desorption isotherm, resulting in hysteresis closure at the lower closure point [44,48]. The pore size distributions corresponding to the N_2_ adsorption isotherms are presented in Figure 2b. ALG beads exhibited meso-macroporous dimensions ranging from 13.2 to 100 nm (a peak at 61.1 nm). In comparison to ALG beads, BCAC/ALG and BCAC/MT/ALG beads had smaller pores. BCAC/ALG beads showed a lower mesoporous range of 3.0 to 15.5 nm (a peak at 10.1 nm), while BCAC/MT/ALG beads had a range of 3.1 to 15.5 nm (a peak at 9.0 nm). The inclusion of BCAC and MT contributed to lower mesoporous ranges, which significantly enhanced the BET surface area of pristine ALG beads.

#### 2.1.4. FE-SEM Analysis

**Figure 3:**
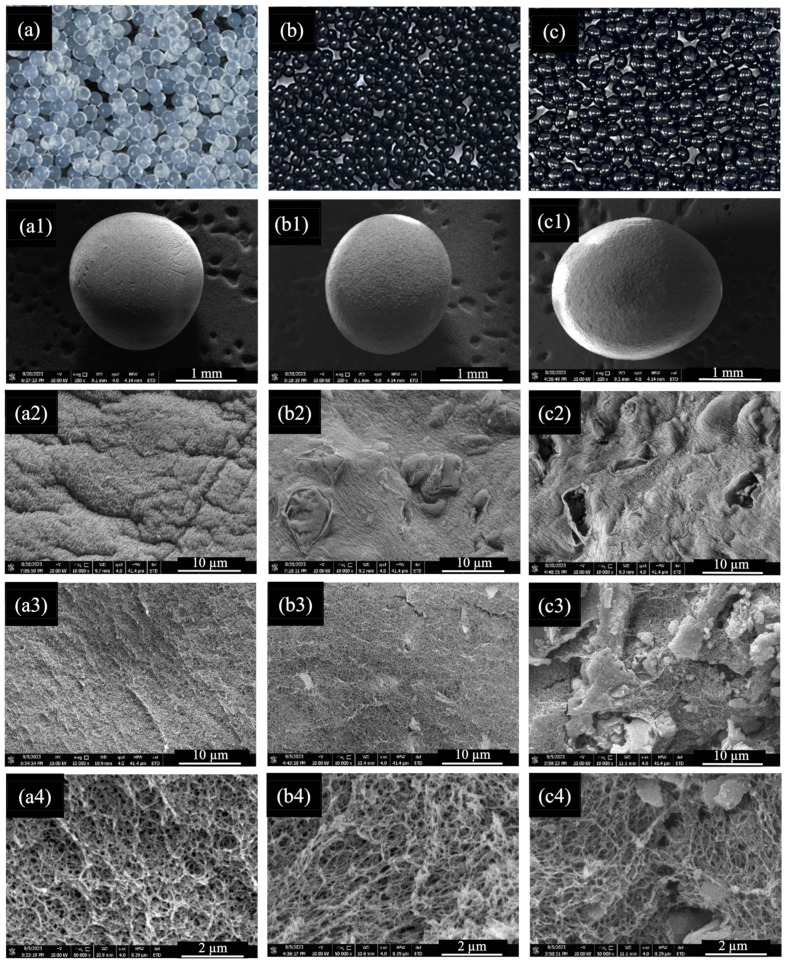
FE-SEM images of adsorbent hydrogel beads ((**a**). ALG, (**b**). BCAC/ALG, (**c**). BCAC/MT/ALG): surface morphologies of ALG (**a1**,**a2**), BCAC/ALG (**b1**,**b2**), and BCAC/MT/ALG (**c1**,**c2**); cross-section morphologies of ALG (**a3**,**a4**), BCAC/ALG (**b3**,**b4**), and BCAC/MT/ALG (**c3**,**c4**).

**Figure 4:**
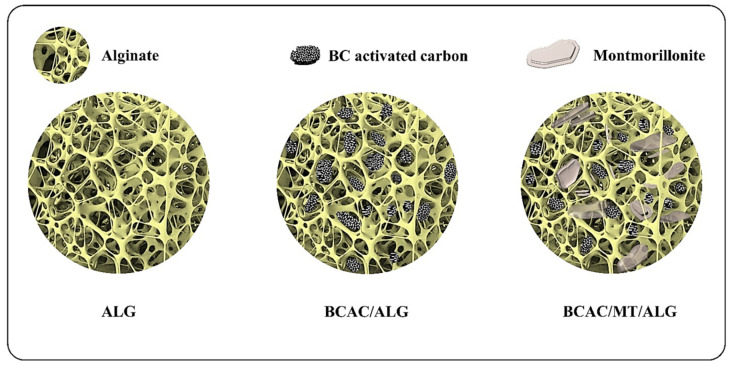
Schematic illustration of the integration of BCAC and MT into the ALG matrix according to cross-section morphologies observed in FE-SEM images.

The surface morphological features were examined using field-emission scanning electron microscopy (FE-SEM). In Figure 3(a1–a2), the alginate beads (ALG) are depicted, revealing a surface micrograph characterized by a distinct texture resembling that of a smooth, soft fabric-like structure. The surfaces of BCAC/ALG and BCAC/MT/ALG beads are shown in Figure 3(b1–b2) and Figure 3(c1–c2), respectively. BCAC incorporation increased surface roughness with numerous protrusions. Additionally, BCAC/MT incorporation with ALG further intensified the surface roughness due to the presence of abundant granular and flaky MT and BCAC particles. Furthermore, a detailed observation of the cross-section of each hydrogel bead revealed a 3D porous dense structure characterized by an interconnected porous network for ALG beads, as depicted in Figure 3(a3–a4). In the case of BCAC/ALG, the BCAC particles exhibited effective dispersion within the porous network of ALG without any discernible aggregates, as observed in Figure 3(b3–b4). Additionally, BCAC/MT incorporation into ALG displayed distinctive plate-like layered structures that were stacked within the porous framework of ALG, as shown in Figure 3(c3–c4). The intrinsic structure of ALG as an encapsulating agent was still visible despite these structural alterations. Overall, the hydrogel beads of ALG and BCAC/ALG are in a spherical shape with a diameter of ~3.7 and ~3.6 mm, respectively. BCAC/MT/ALG is slightly larger than ALG and BCAC/ALG with a diameter of ~3.9 mm. When BCAC and montmorillonite clay were introduced into the alginate solution, sonication was employed to enhance dispersion and minimize bubble formations in the gel solution. Additionally, to achieve uniform and similar shapes of hydrogel beads, a burette was used to control the droplet formation. This approach helped maintain consistency in shape and distribution. The schematic diagram illustrating the combination and porous structures of composite hydrogel beads is shown in Figure 4.

### 2.2. Comparison of Adsorption Uptakes of Adsorbents for Removal of MB

**Figure 5:**
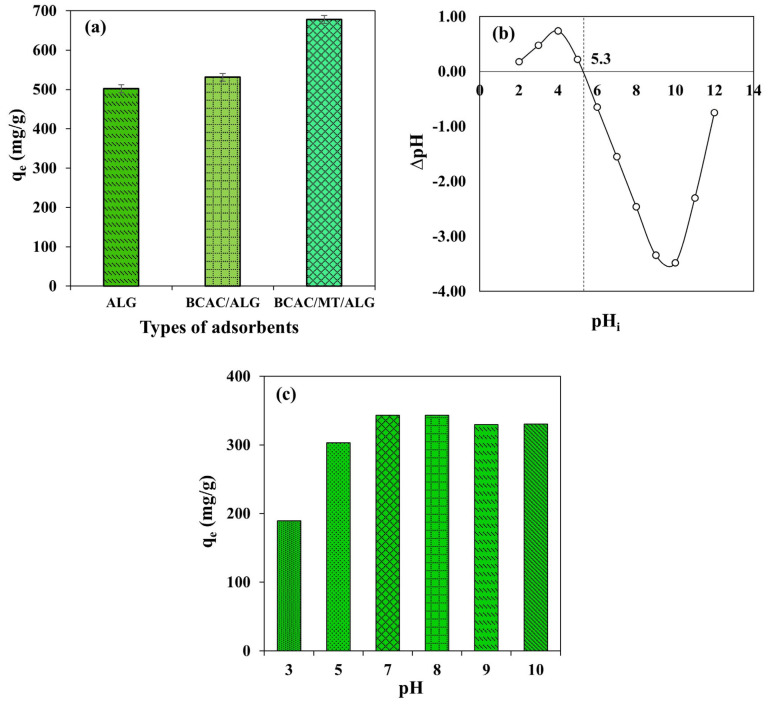
Comparison of MB adsorption uptakes on various adsorbent hydrogel beads (**a**), pH at the point of zero charge of BCAC/MT/ALG beads (**b**), and effect of solution pH on MB adsorption using BCAC/MT/ALG beads (**c**). Data are available in the supplementary materials (Tables S1–S3).

Adsorption uptakes of adsorbents (ALG, BCAC/ALG, and BCAC/MT/ALG) for removal of MB were conducted at an initial MB concentration of 600 mg/L, 150 rpm, pH 7, and 30 °C. The adsorption capacities were compared as shown in Figure 5a. The MB adsorption uptakes at equilibrium (*q_e_*) of ALG, BCAC/ALG, and BCAC/MT/ALG beads were at 501.85 mg/g, 530.81 mg/g, and 678.16 mg/g, respectively. The adsorbent of BCAC/MT/ALG bead exhibited superior MB uptake with ~1.4 and ~1.3 times higher adsorption uptake of MB than that of ALG and BCAC/ALG beads, respectively, attributed to its high specific surface area with an appropriate mesoporous structure [40,49]. Given the molecular size of MB at 1.38 nm, the mesoporous structure of BCAC/MT/ALG exhibits a pronounced capability to adsorb larger molecules like MB, a function of which micropores exhibit inherent limitations [4,21]. This elucidation highlights the significance of tailored adsorbent structures in accommodating the unique characteristics of the target adsorbate. Although the specific surface area and porosity of ALG could be increased for example, by lower sodium alginate concentration [50], the mechanical strength of the obtained alginate bead would be weaker and not suitable for practical applications. In addition, the surface functional groups of BCAC and the probability of ionic exchange of MT might also enhance the adsorption uptake of MB in the aqueous solution [36]. Since BCAC/MT/ALG beads exhibited the highest MB adsorption uptake, further studies of kinetic models, adsorption isotherms, and reusability were focused only on BCAC/MT/ALG beads.

### 2.3. Effect of pH

The pH level is a critical parameter in adsorption, influencing the adsorbent’s surface charge, reactivity or dissociation of functional groups, and the ionization behavior of sorbate molecules interacting with the adsorbent [6,9]. The point of zero charge (pH_pzc_) for BCAC/MT/ALG beads is 5.3, as depicted in Figure 5b. The influence of the pH range between 3 and 10 on the adsorption uptake with an initial MB concentration of 200 mg/L for 12 h at 30 °C is illustrated in Figure 5c. A decrease in the adsorption of MB was observed at pH levels below 5.3. This phenomenon is attributed to the competitive adsorption between cationic methylene blue (MB^+^) and hydronium ions (H_3_O^+^) in the solution. Notably, hydronium ions exhibit a greater affinity for adsorption compared to MB^+^ [51]. Furthermore, at lower pH levels, both carboxyl (−COOH) and hydroxyl (−OH) groups predominantly exist in a non-ionized state. This non-ionized state was crucial as it contributed to the positively charged surface of hydrogel beads, resulting in the generation of repulsive forces that impede the adsorption process. In contrast, the increase in MB adsorption beyond a pH of 5.3 could be attributed to the higher density of hydroxide ions (OH^−^) and the ionization of most carboxyl groups into carboxylate ions (−COO^−^) within the composite hydrogel beads, which promoted electrostatic attraction between MB^+^ molecules and the negatively charged surface of the BCAC/MT/ALG beads, thus facilitating the adsorption process [9,33,49]. Nonetheless, no substantial increment in adsorption uptake was observed in the transition from pH 7 to 8. Thus, pH 7 was selected as the optimum pH for further studies.

### 2.4. Effect of Initial MB Concentration and Contact Time

**Figure 6:**
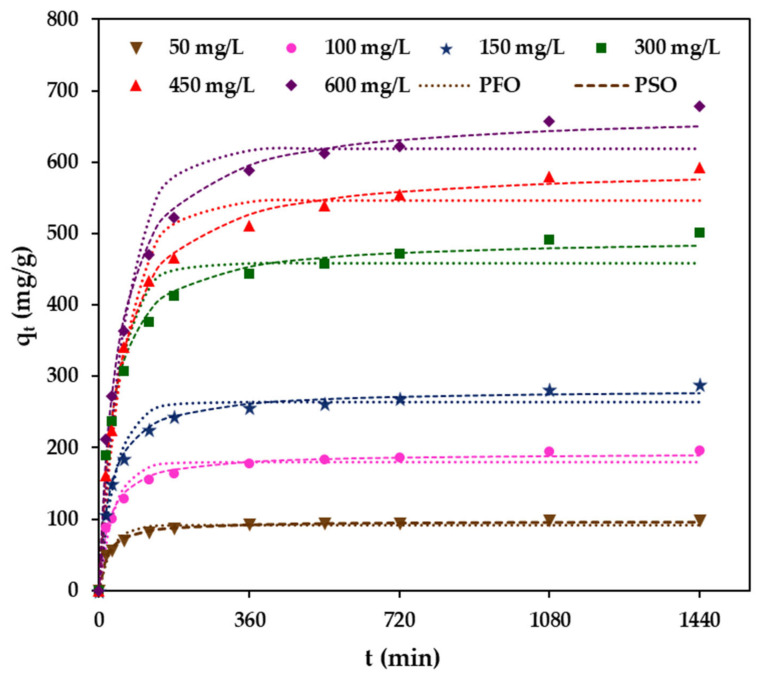
Effect of initial concentrations of MB and contact time on the adsorption capacities (*q_t_*) of BCAC/MT/ALG hydrogel beads. Experimental data were compared with calculated data from the pseudo-first order (PFO) and pseudo-second order (PSO) kinetic models. Data are available in the supplementary materials (Table S4).

The effect of initial MB concentration on the adsorption uptake using BCAC/MT/ALG beads under the conditions of 150 rpm, pH 7, and 30 °C is presented in Figure 6. The adsorption uptake of MB exhibited an upward trend as the initial concentrations of MB increased from 50 mg/L to 600 mg/L. The adsorption process displayed a rapid initial adsorption uptake within the first 180 min, followed by a gradual increase in adsorption uptake, reaching equilibrium at approximately 1440 min. As the MB concentration increased from 50 to 600 mg/L, the adsorption uptake rose significantly from 97.8 to 678.2 mg/g, driven by the higher number of MB molecules available at the higher MB concentration, allowing more adsorbate molecules to bind to vacant sites on the composite beads. Furthermore, elevated driving forces at higher MB concentrations facilitate the rapid mass transfer of MB from the aqueous phase into hydrogel beads [21,33]. The maximum adsorption uptake of BCAC/MT/ALG (*q_m_* = 719.17 mg/g) places it as one of the most effective developed adsorbents for MB adsorption, in addition to adsorbents such as Pd-ZnO@H^+^-Mt (*q_m_* = 618.14 mg/g) [40], AC-alginate beads (*q_m_* = 287.35 mg/g) [4], *Dodonaea viscosa*-activated carbon-alginate beads (*q_m_* = 370 mg/g) [6], porous cellulose-derived carbon/montmorillonite (*q_m_* = 138.1 mg/g) [44], and SA/CMC-MeS (*q_m_* = 230 mg/g) [41]. However, the maximum adsorption capacity may also depend on various operating parameters, including pH, dosage, contact time, temperature, coexisting ions, and applied adsorption kinetics and isotherm.

### 2.5. Adsorption Kinetics

The adsorption kinetics of BCAC/MT/ALG beads for the removal of MB in an aqueous solution were assessed by examining the effect of contact time on the adsorption process across various initial concentrations of MB. Based on the adsorption kinetic parameters provided in Table 3 and the nonlinear fitted plots illustrated in Figure 6, it is evident that the adsorption kinetics of MB onto BCAC/MT/ALG beads, especially at high concentrations of MB (≥300 mg/L), could be more accurately described by the PSO kinetic model. The preference for the PSO kinetic model is supported by consistently higher correlation coefficient (*R*^2^) values, which outperform the PFO kinetic model. Furthermore, the theoretically calculated adsorption uptakes (*q_cal_*) from PSO closely align with the experimentally obtained adsorption uptakes (*q_exp_*) values. These findings align with previous studies involving a range of alginate composites [6,21,41].

### 2.6. Equilibrium Isotherm Models

**Figure 7:**
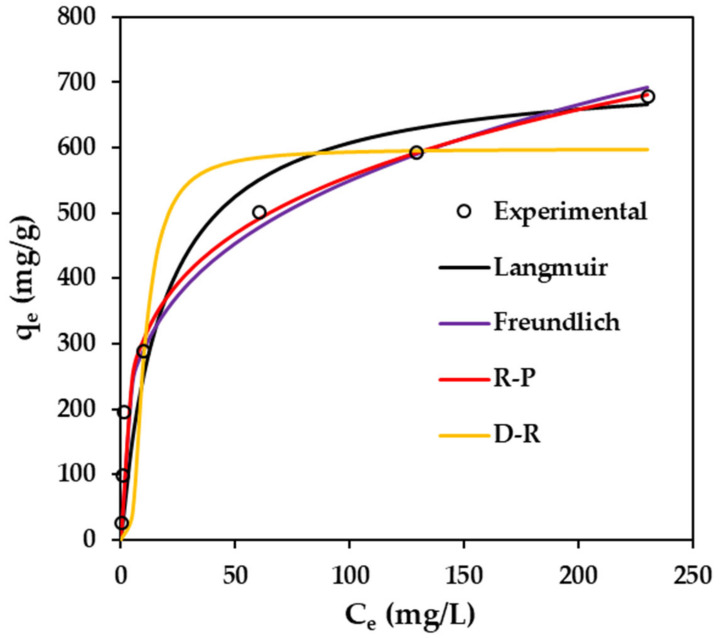
Comparison between experimental data and calculated data from the equilibrium isotherm models for MB adsorption on BCAC/MT/ALG hydrogel beads at 30 °C. Data are available in the supplementary materials (Table S5).

The adsorption isotherm defines interactions between the adsorbent and adsorbate, offering valuable insights into the intrinsic properties of the adsorption process. In this study, the equilibrium experimental data were correlated using empirical models, comprising the non-linear forms of the Langmuir, Freundlich, Redlich-Peterson, and Dubinin-Radushkevich isotherm models, as illustrated in Figure 7. The relevant parameters and the correlation coefficients (*R*^2^) for these non-linear isotherms were determined, as provided in Table 4. The Langmuir isotherm [52] describes the phenomenon of monolayer adsorption on a uniform and homogeneous surface, where each adsorbate molecule on the surface possesses equivalent adsorption energy, with no interactions occurring between adsorbate molecules on adjacent sites. In contrast, the Freundlich model [53] proposes a multilayer adsorption process on a heterogeneous surface, characterized by intermolecular interactions among adsorbed molecules and exponentially distributed binding affinities at adsorption sites, reflecting varying affinities for adsorption. In this study, compared to all the applied isotherm models, the Redlich-Peterson model [54] shows the best fit to interpret the adsorption equilibrium across a broad spectrum of MB concentrations and has the highest correlation coefficient value (*R*^2^ = 0.994). The acquired *b_RP_* value lies between 0 and 1 (0 < *b_RP_* = 0.7634 < 1), signifying that the adsorption process of MB onto BCAC/MT/ALG beads exhibits characteristics of both Freundlich and Langmuir models, indicating adsorption occurrences on both homogeneous and heterogeneous surfaces. Additionally, the Dubinin-Radushkevich model [55] predicts that the MB adsorption on BCAC/MT/ALG hydrogel beads is physical adsorption, wherein the free energy (*E* = 3.8801 kJ/mol) is less than 8 kJ/mol. This observation suggests that the interaction between the absorbent and the adsorbate is primarily governed by non-covalent forces, such as electrostatic interactions and hydrogen bonding, as supported by the FTIR spectra, rather than chemical bonding.

### 2.7. Reusability of the Hydrogel Beads

**Figure 8:**
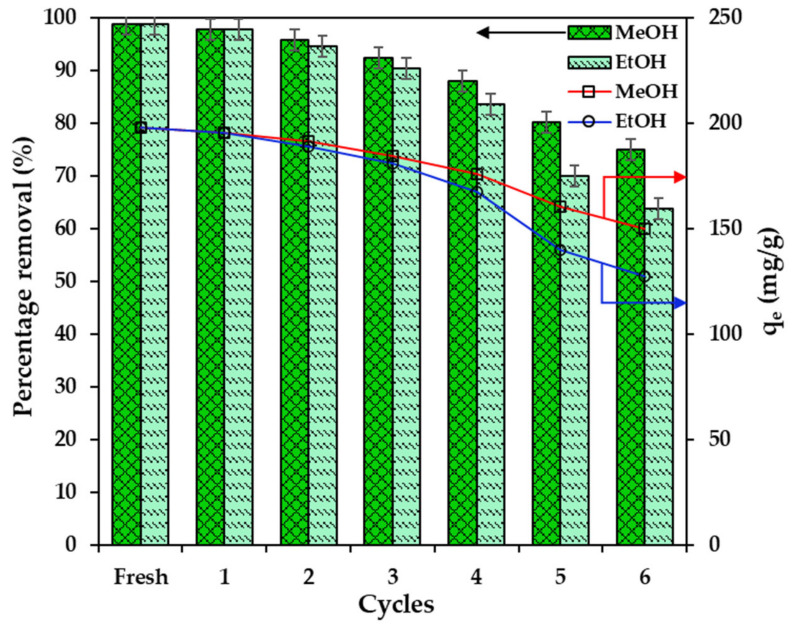
Reusability of BCAC/MT/ALG hydrogel beads for MB removal. Data are available in the supplementary materials (Tables S6 and S7).

The reusability of adsorbents is crucial for determining the practical viability of wastewater treatment technologies. Industrial acceptance requires economic feasibility through highly reusable materials. Therefore, a cyclic adsorption-desorption experiment of BCAC/MT/ALG beads was repeated six times with an initial MB concentration of 100 mg/L. Ethyl alcohol (EtOH) and methyl alcohol (MeOH) were used as the desorbing agents. Figure 8 presents the percentage removal and adsorption uptake for MB through six adsorption-desorption cycles. The outcomes reveal consistent percentage removal, with 98.86% for fresh adsorption and 97.83% and 97.76% for the first cycle using MeOH and EtOH, respectively. However, by the sixth cycle, MeOH and EtOH achieved lower percentages of removals at 75.07% and 63.71%, respectively. This highlights MeOH’s superior efficacy as a desorbing agent, consistently yielding higher percentage removals throughout the cycles. The decline in percentage removal may be ascribed to fewer vacant binding sites and partial occupancy caused by incomplete MB desorption from the hydrogel beads [41]. However, no substantial decline in percentage removal was observed over six cycles, demonstrating the remarkable reusability of BCAC/MT/ALG hydrogel beads.

## 3. Conclusions

The novel hydrogel bead of BCAC/MT/ALG was effectively fabricated through an ionotropic gelation method, with calcium chloride serving as a cross-linking agent. The BCAC and MT incorporation into pristine alginate beads (ALG) resulted in a substantial enhancement of the BET surface area to 335.5 m^2^/g. The reinforcement was associated with a unified pore network featuring a meso-macroporous structure intertwined with aggregates composed of plate-like particles of MT and BCAC. Furthermore, the additions of BCAC and MT significantly enhanced the thermal stability and mechanical properties of BCAC/MT/ALG beads as compared to pristine ALG beads. At pH 7 and 30 °C, the BCAC/MT/ALG bead displayed an outstanding capacity for MB adsorption of 678.2 mg/g. The reusability evaluation demonstrated the BCAC/MT/ALG bead’s durability and ability to be reused for at least six consecutive cycles with greater than 75% MB removal by using methanol as a desorbing agent. Thus, BCAC/MT/ALG bead has a good potential to be used as a highly efficient, cost-effective, and environmentally friendly adsorbent for removing dye from aqueous solutions.

## 4. Materials and Methods

### 4.1. Chemicals

Bacterial cellulose (BC), which served as the precursor for activated carbon (AC), was purchased from a local manufacturer (Bangkok, Thailand). Analytical reagent-grade chemicals, including ortho-phosphoric acid (85%, RCI Labscan, Bangkok, Thailand), montmorillonite (K10, Sigma-Aldrich, Darmstadt, Germany), sodium alginate (ACROS Organics, Shanghai, China), calcium chloride (93%, Ajax Finechem, Sydney, Australia), methylene blue (C_16_H_18_N_3_ClS·2H_2_O, Ajax Finechem, Sydney, Australia), sodium hydroxide (97%, KemAus, Melbourne, Australia), hydrochloric acid (37%, QRëC, Auckland, New Zealand), methyl alcohol (99.8%, J.T.Baker, Phillipsburg, New Jersey, USA), and ethyl alcohol (99.9%, QRëC, Auckland, New Zealand), were used without additional processing. Deionized water was used for the preparation of all necessary solutions.

### 4.2. Preparation of BCAC

Bacterial cellulose (BC) was utilized as a precursor material in the synthesis of BCAC. The preparation of BCAC was based on the procedure previously reported by Khamkeaw et al. 2018 [36]. BC was initially treated with a 1% (*w*/*v*) sodium hydroxide solution for 24 h to remove residual bacterial cells. Subsequently, it was rinsed with deionized water until it reached a neutral pH and then dried at 110 °C for 24 h in an electric oven (58/350 LSN11, SNOL, Utena, Lithuania) [56]. The chemical activation was performed using concentrated H_3_PO_4_ with a 1:1 ratio (*w/w*) of BC to H_3_PO_4_ at 30 °C for 24 h and then dried in an oven at 110 °C for 24 h. Thereafter, H_3_PO_4_-impregnated BC products were carbonized at 500 °C for 1 h in a muffle furnace (CWF 1100, Carbolite, Derbyshire, UK). The obtained BCAC was washed with a 1 M HCl solution, followed by repeatedly rinsing with deionized water until it reached a neutral pH, and dried at 110 °C for 24 h. Previously, the characteristics and properties of BCAC have been thoroughly characterized and reported [36].

### 4.3. Preparation of BCAC/MT/ALG Hydrogel Beads

BCAC/MT/ALG hydrogel beads were prepared by dissolving 2% (*w*/*v*) sodium alginate into deionized water and stirring at 40 °C for 2 h. Thereafter, BCAC and MT were added to a sodium alginate solution with a 1:1:1 ratio (*w*/*w*/*w*) of BCAC/MT/ALG under vigorous stirring at 40 °C for 3 h. The mixture was then sonicated for 10 min using an ultrasonic cleaner (CREST Ultrasonics 950HT, Penang, Malaysia). The mixture was then transferred into a 50-mL burette and dropped into a 0.5 M CaCl_2_ solution under mild stirring. The formed hydrogel beads were kept overnight to facilitate cross-linking and ensure complete gelation, then rinsed with deionized water to remove unbounded CaCl_2_ from the surface of the hydrogel beads. The obtained BCAC/MT/ALG hydrogel beads were stored in deionized water at 5 °C for further use. ALG and BCAC/ALG hydrogel beads (1:1 ratio (*w*/*w*) of BCAC:ALG) were also prepared following an identical procedure.

### 4.4. Characterization

Surface morphologies were analyzed via FE-SEM using a QUANTA FEG 250 (Thermo Fisher FEI, Waltham, MA, USA). Surface functional groups were identified using FT-IR spectroscopy performed with a Bruker INVENIO-S FTIR (Bruker Corporation, Billerica, MA, USA); the recorded spectra were obtained over a wavelength range from 4000 to 400 cm^−1^. N_2_ physisorption was assessed at −196 °C using an Autosorb iQ Station 2 (Quantachrome Instruments, Boynton Beach, FL, USA), measuring specific surface area (BET method), pore size distribution (BJH method), micropore volume (Harkins-Jura standard isotherm), and total pore volume. Thermal degradation patterns were analyzed via TGA using a NETZSCH STA 449 F3 Jupiter (NETZSCH-Geratebau GmbH, Selb, Germany) between 30 and 800 °C with a heating rate of 5 °C/min under a N_2_ atmosphere. Mechanical properties were evaluated using a Hounsfield UTM H10 KM (Hounsfield Test Equipment, Redhill, UK) following ASTM D882 standards. The point of zero charge (pH_pzc_) was determined following a previously reported method [57]. Briefly, a series of Erlenmeyer flasks, each containing 30 mL of a 0.1 M NaCl solution, was adjusted to a pH value ranging from 3 to 10 using either a 0.1 M HCl or NaOH solution. An amount of 0.025 g of adsorbent was added to each Erlenmeyer flask at varying pH values and shaken for 24 h at 25 °C until reaching equilibrium. The final pH (pH_f_) was then measured to plot (pH_f_ − pH_i_) against pH_i_ to determine the pH_pzc_.

### 4.5. Adsorption of Methylene Blue

Methylene blue was employed as a representative cationic dye model in a batch adsorption experiment using adsorbent beads. The influences of adsorption parameters, including adsorbent types (BCAC/MT/ALG; BCAC/ALG; ALG beads), initial MB concentrations (50 to 600 mg/L), pH (3 to 10), and contact time (15 to 1440 min), were studied. The adsorption procedure was conducted by adding 0.025 g of adsorbent into each Erlenmeyer flask containing 50 mL of the MB solution at 150 rpm and 30 °C in an incubator shaker (Innova 4330, New Brunswick Scientific, Edison, New Jersey, USA). MB concentrations were determined by using a UV-Vis spectrometer (UV-2450, Shimadzu, Kyoto, Japan) at λ = 664 nm. The adsorption uptake per gram of adsorbent at equilibrium (*q_e_*, mg/g) and percentage of removal (*R*%) were evaluated using Equations (1) and (2), respectively.

(1) $$q_{e}=\frac{\left(C_{0}-C_{e}\right)}{m}\times V$$

(2) $$R\%=\frac{\left(C_{0}-C_{e}\right)}{C_{0}}\times 100$$

where *C*_0_ and *C_e_* are the initial and equilibrium concentrations of MB (mg/L), respectively, *m* is the mass of adsorbent (g), and *V* is the volume of MB solution (L).

### 4.6. Kinetic Model Study

In a series of experiments, batch adsorption tests were performed at various time intervals to assess the adsorption behavior of MB onto the BCAC/MT/ALG hydrogel beads. The primary objective was to understand the underlying adsorption mechanisms. This involved analyzing data collected at different time points using two established kinetic models: the pseudo-first-order (PFO) model [58] and the pseudo-second-order (PSO) model [59]. The non-linear forms of PFO and PSO kinetic models are presented in Equation (3) and Equation (4), respectively.

(3) $$q_{t}=q_{e}\;\left(1-e^{-k_{1}t}\right)$$

(4) $$q_{t}=\frac{q_{e}^{2}k_{2}t}{1+q_{e}k_{2}t}\;$$

where *q_e_* is MB adsorption uptake at equilibrium (mg/g), *q_t_* is MB adsorption uptake at a given time *t* (mg/g), *k*_1_ is the adsorption rate constant (1/min) in Equation (3), and *k*_2_ is the adsorption rate constant (g/mg.min) in Equation (4). Additionally, *q_t_._exp_* is the experimental adsorption uptake at time *t* (mg/g); *q_t_._cal_* is the calculated adsorption uptake at time *t* determined by the models (mg/g); and *n* is the number of observations.

### 4.7. Adsorption Isotherm Study

Adsorption isotherm analysis was undertaken to evaluate the interaction between the adsorbent and the adsorbate (MB). Adsorption isotherms offer valuable insights into the nature of adsorption processes. Within this framework, the non-linear forms of the Langmuir [52], Freundlich [53], Redlich-Peterson [54], and Dubinin-Radushkevich [55] isotherm models were employed to analyze and comprehend this behavior, represented in Equations (5)–(8), respectively.

(5) $$q_{e}=\frac{q_{m}K_{L}C_{e}}{1+K_{L}C_{e}}\;$$

(6) qe=KFCe1n

(7) $$q_{e}=\frac{K_{RP}C_{e}}{1+a_{RP}C_{e}^{b_{RP}}}\;$$

(8) $$q_{e}=q_{D}.exp\left(-\beta \times \varepsilon^{2}\right)$$

where *q_e_* is the adsorption uptake at equilibrium (mg/g), *q_m_* is the maximum monolayer adsorption uptake (mg/g), *K_L_* is Langmuir’s constant associated with the adsorption energy (L/mg), *K_F_* is the Freundlich adsorption constant, *n* is the exponential coefficient associated with adsorption intensity, *K_RP_* is the Redlich-Peterson (R-P) isotherm constant (L/g), *a_RP_* is the R-P isotherm constant (L/mg), *b_RP_* is the R-P isotherm exponent (0 < *b_RP_* < 1), *q_D_* is the theoretical monolayer saturation uptake (mg/g), *β* is the D-R constant (mol^2^/kJ^2^) related to the free energy (*E*), where *E =* 1/√2*β* (kJ/mol), and *ε* is Polanyi potential (*ε* = *RTln*(1 + 1/*C_e_*)).

### 4.8. Reusability Study

The reusability study of BCAC/MT/ALG beads was conducted using methyl alcohol and ethyl alcohol as the eluting agents to desorb MB from spent BCAC/MT/ALG beads. Initially, 0.025 g of spent BCAC/MT/ALG beads were washed with deionized water, followed by rinsing and magnetically stirring with 25 mL of methyl alcohol or ethyl alcohol as a desorbing agent. The hydrogel beads were then further rinsed with deionized water until neutral. Reusability was evaluated through six cycles of adsorption-desorption experiments with an initial MB concentration of 100 mg/L.

## Figures and Tables

**Table 1:** Mechanical properties (tensile strength, Young’s modulus, and elongation at break) of adsorbent hydrogel beads of ALG, BCAC/ALG, and BCAC/MT/ALG.

Sample	Tensile Strength (MPa)	Young’s Modulus (MPa)	Elongation (%)
ALG	7.39 ± 2.75	31.87 ± 15.73	89.46 ± 14.85
BCAC/ALG	15.72 ± 2.59	141.14 ± 35.32	19.71 ± 3.50
BCAC/MT/ALG	9.08 ± 0.44	69.90 ± 7.08	30.17 ± 2.99

**Table 2:** Specific surface area and porous properties of adsorbent hydrogel beads of ALG, BCAC/ALG, and BCAC/MT/ALG.

Sample	^1^ *S_BET_* (m^2^/g)	Pore Volume (cm^3^/g)			^5^ *D_p_* (nm)
		^2^ *V_micro_*	^3^ *V_meso_*	^4^ *V_T_*	
ALG	287.0	0.010	4.820	4.830	67.3
BCAC/ALG	387.9	0.036	2.484	2.520	25.9
BCAC/MT/ALG	335.5	0.018	3.622	3.640	43.4

^1^ BET surface area; ^2^ Micropore volume was calculated by the t-plot method; ^3^ Mesoporous volume was calculated by *V_T_*–*V_micro_*; ^4^ Total pore volume was calculated at the relative pressure of 0.99; ^5^ Average pore size diameters.

**Table 3:** The values of adsorption uptakes (*q_exp_*) from the experimental study at various initial MB concentrations from 50–600 mg/L using BCAC/MT/ALG hydrogel beads at 30 °C and the fitting parameters of the pseudo-first-order and pseudo-second-order kinetic models. The pseudo-second-order model was found to be the best representative of the adsorption kinetics of BCAC/MT/ALG beads for the removal of MB in an aqueous solution.

*C*_0_ (mg/L)	*q_exp_* (mg/L)	Pseudo-First-Order			Pseudo-Second-Order		
		*k*_1_ (1/min)	*q_cal_* (mg/g)	*R^2^*	*k_2_* (g/mg.min)	*q_cal_* (mg/g)	*R^2^*
50	97.76	1.9791	91.75	0.951	0.0317	97.12	0.991
100	195.64	1.6265	180.47	0.943	0.0127	192.43	0.988
150	288.48	1.4826	263.62	0.962	0.0077	282.14	0.995
300	501.78	1.2880	459.14	0.953	0.0038	493.09	0.992
450	592.74	0.9425	546.25	0.973	0.0022	594.64	0.997
600	678.16	0.9219	618.48	0.956	0.0020	670.88	0.992

**Table 4:** The values of fitting parameters of equilibrium isotherm models for MB adsorption on BCAC/MT/ALG hydrogel beads at 30 °C. Redlich-Peterson isotherm was found to be the best representative (*R*^2^ = 0.994) for this adsorption system.

Isotherm Model	Parameter	Value	*R* ^2^
Langmuir	*q_m_* (mg/g)	719.17	0.921
	*K_L_* (L/mg)	0.0541	
Freundlich	*n*	3.6094	0.991
	*K_F_*	153.46	
Redlich-Peterson (R-P)	*K_RP_* (L/g)	518.92	0.994
	*a_RP_* (L/mg)	2.7434	
	*b_RP_*	0.7634	
Dubinin-Radushkevich (D-R)	*q_D_* (mg/g)	597.97	0.839
	*β* (mol^2^/kJ^2^)	0.0332	
	*E* (kJ/mol)	3.8801	

## Data Availability

The original contributions presented in the study are included in the article. Further inquiries can be directed to the corresponding author.

## Supplementary Materials

The following supporting information can be downloaded at: https://www.mdpi.com/article/10.3390/gels10090597/s1, Table S1: Adsorption uptake of various adsorbent hydrogel beads; Table S2: Data for pH at the point of zero charge of BCAC/MT/ALG beads; Table S3: Effect of solution pH on MB adsorption using BCAC/MT/ALG beads; Table S4: Experimental data for the effect of initial concentrations of MB and contact time on the adsorption capacities (q_t_) (mg/g) of BCAC/MT/ALG hydrogel beads; Table S5: Experimental data and adsorption isotherm data at equilibrium according to their equations; Table S6: Reusability of BCAC/MT/ALG hydrogel beads for MB removal by using methanol as a desorbing agent; Table S7: Reusability of BCAC/MT/ALG hydrogel beads for MB removal by using ethanol as a desorbing agent.
