# Supplementary material for: Alginate-Based Hydrogel Bead Reinforced with Montmorillonite Clay and Bacterial Cellulose-Activated Carbon as an Effective Adsorbent for Removing Dye from Aqueous Solution
Source: Gels. 2024 Sep 16;10(9):597. doi: 10.3390/gels10090597 (PMC11431803; doi:10.3390/gels10090597)
Supplement: Supplementary file 1 [file gels-10-00597-s001.zip › gels-3201818-supplementary.pdf]

Supplementary materials

Alginate-Based Hydrogel Bead Reinforced with Montmorillonite Clay and Bacterial Cellulose-Activated Carbon as an Effective Adsorbent for Removing Dye from Aqueous Solution

Muhammad Dody Isnaini, Bhawaranchat Vanichsetakul and Muenduen Phisalaphong *

Bio-Circular-Green-economy Technology & Engineering Center, BCGeTEC, Department of Chemical Engineering, Faculty of Engineering, Chulalongkorn University, Bangkok, 10330, Thailand; muhammad.dody06@gmail.com (M.D.I.); 6671009121@student.chula.ac.th (B.V.)

* Correspondence: muenduen.p@chula.ac.th

Table S1. Adsorption uptake of various adsorbent hydrogel beads.

Adsorbent type	MB uptake (q_e) (mg/g)
ALG	501.8544
BCAC/ALG	530.8136
BCAC/MT/ALG	678.1558

Table S2. Data for pH at the point of zero charge of BCAC/MT/ALG beads.

pH_i	pH_f	ΔpH ($pH_f - pH_i$)
2	2.18	0.18
3	3.48	0.48
4	4.74	0.74
5	5.22	0.22
6	5.35	−0.65
7	5.45	−1.55
8	5.54	−2.46
9	5.66	−3.34
10	6.52	−3.48
11	8.70	−2.30
12	11.25	−0.75

Table S3. Effect of solution pH on MB adsorption using BCAC/MT/ALG beads.

pH	MB uptake (q_e)
3	189.5330
5	303.0210
7	343.0639
8	343.1963
9	329.4182
10	330.2793

Table S4. Experimental data for the effect of initial concentrations of MB and contact time on the adsorption capacities (q_t) (mg/g) of BCAC/MT/ALG hydrogel beads.

contact time (min)	50 mg/L	100 mg/L	150 mg/L	300 mg/L	450 mg/L	600 mg/L
15	48.9441	88.3659	106.9798	188.7878	161.1734	211.3071
30	55.5682	101.2830	150.2353	236.9784	224.4339	272.7262
60	70.0088	128.9719	185.2108	308.0222	341.3499	364.0592
120	81.4851	155.7003	226.1479	376.9132	433.7565	470.3493
180	86.6851	164.5104	243.4369	413.0147	465.5524	522.8578
360	92.5077	177.6924	256.2877	444.1481	511.2589	587.7775
540	93.8060	183.0579	262.4482	458.2244	538.7490	611.9632
720	94.3558	185.8069	268.8073	471.4727	555.3094	621.8285
1080	97.4857	194.7595	281.7244	490.8483	579.8187	656.8342
1440	97.7639	195.6405	288.4810	501.7781	592.7357	678.1558

Table S5. Experimental data and adsorption isotherm data at equilibrium according to their equations.

Experimental data			Langmuir	Freundlich	R-P	D-R
C_i (mg/L)	C_e (mg/L)	q_e (mg/g)	$q_e = \frac{q_m K_L C_e}{1 + K_L C_e}$	$q_e = K_F C_e^{1/n}$	$q_e = \frac{K_{RP} C_e}{1 + a_{RP} C_e^{b_{RP}}}$	$q_e = q_D . exp(-\beta . \varepsilon^2)$
0.5	0.5	25	0.5	0.5	0.5	0
50	0.6955	97.7639	26.0821	138.7775	117.2129	0.000000
100	1.5418	195.6405	55.3721	173.0202	166.0594	0.000001
150	10.1018	288.4810	254.1568	291.2584	307.7209	288.286357
300	60.4452	501.7781	550.7631	478.1226	491.3392	584.933752
450	129.3362	592.7357	629.2507	590.2912	592.3008	595.074968
600	230.5536	678.1558	665.7986	692.8214	681.2461	597.056066

Table S6. Reusability of BCAC/MT/ALG hydrogel beads for MB removal by using methanol as a desorbing agent.

Cycle	Contact time (h)	MB uptake (q_e) (mg/g)	%R
Fresh	24	197.7535	98.86
1	24	195.7034	97.83
2	24	191.6495	95.81
3	24	184.6266	92.30
4	24	176.0710	88.02
5	24	160.5622	80.27
6	24	150.1787	75.07

Table S7. Reusability of BCAC/MT/ALG hydrogel beads for MB removal by using ethanol as a desorbing agent.

Cycle	Contact time (h)	MB uptake (q_e) (mg/g)	%R
Fresh	24	197.7535	98.86
1	24	195.5674	97.76
2	24	189.0984	94.53
3	24	180.8381	90.40
4	24	167.3960	83.68
5	24	140.1933	70.08
6	24	127.4545	63.71
